# Decolonizing the exploration of perinatal mental health screening with Indigenous Australian parents in primary care

**DOI:** 10.1017/S1463423624000665

**Published:** 2025-07-30

**Authors:** Jayne Kotz, Corinne Reid, Melanie Robinson, Roz Walker, Tracy Reibel, Alison Bairnsfather-Scott, Rhonda Marriott

**Affiliations:** 1 Ngangk Yira Institute for Change, Murdoch University, Murdoch, WA, Australia; 2 Global Health Academy and Academy of Sport, The University of Edinburgh, Edinburgh, United Kingdom; 3 Aboriginal Health Child and Adolescent Health Service, WA, Australia; 4 School of Indigenous Knowledges, Murdoch University, Murdoch, WA, Australia; 5 Kids Institute Australia, Nedlands, Australia

**Keywords:** Australian Aboriginal, codesign, decolonising research, Indigenous, participatory action research, perinatal mental health

## Abstract

**Background:**

Effective mental health primary prevention and early detection strategies targeting perinatal mental healthcare settings are vital. Poor maternal mental health places the developing foetus at risk of lasting cognitive, developmental, behavioural, physical, and mental health problems. Indigenous women endure unacceptably poor mental health compared to all other Australians and disproportionately poorer maternal and infant health outcomes. Mounting evidence demonstrates that screening practices with Indigenous women are neither effective nor acceptable. Improved understanding of their perinatal experiences is necessary for optimizing successful screening and early intervention. Achieving this depends on adopting culturally safe research methodologies.

**Methodology:**

Decolonizing translational research methodologies are described. Perspectives of Australian Indigenous peoples were centred on leadership in decision-making throughout the study. This included designing the research structure, actively participating throughout implementation, and devising solutions. Methods included community participatory action research, codesign, and yarning with data analysis applied through the cultural lenses of Indigenous investigators to inform culturally meaningful outcomes.

**Discussion:**

The Indigenous community leadership and control, maintained throughout this research, have been critical. Allowing time for extensive community collaboration, fostering mutual trust, establishing strong engagement with all stakeholders and genuine power sharing has been integral to successfully translating research outcomes into practice. The codesign process ensured that innovative strengths-based solutions addressed the identified screening barriers. This process resulted in culturally sound web-based perinatal mental health and well-being assessment with embedded potential for widespread cultural adaptability.

## Background

Indigenous^1^ women continue to experience unacceptably poorer pregnancy and birth outcomes overall (ARACY, 2018). They experience the highest rates of psychological distress compared to all other Australians (Australian Bureau of Statistics, 2019). Effective perinatal primary healthcare includes mental health screening and supportive follow-up of maternal physical and mental health and social functioning. In combination, these elements are known to improve maternal and infant outcomes. Whilst the national guidelines provide consensus-based recommendations and practice points based on expert opinion to avoid cultural bias (Austin *et al.*, 2017), the Edinburgh Postnatal Depression Scale (EPDS) has never been validated with Indigenous women (Chan *et al.*, 2021). Such outcomes cannot be viewed as valid or reliable indicators (Dockery *et al.*, 2010). Moreover, psychosocial assessments of Indigenous women are often neglected or of highly variable quality (Gausia *et al.*, 2013). If Indigenous women are flagged as at risk, frequently no further action is taken (Langham *et al.*, 2017).

Mainstream screening/assessment tools, like the EPDS, fail to consider Indigenous peoples lived experiences of mental health as a blend of social, emotional, spiritual, and cultural life (Dockery *et al.*, 2010). Additionally, cultures both interdisciplinary and interracially influence the experience, expression, and communication of symptoms and how these are managed. Applying Western biomedical thinking when translating, interpreting, or proposing management strategies may therefore result in underdiagnosis, overdiagnosis, or misdiagnosis (Parker and Milroy, 2014).

From a primary healthcare perspective, the current inequities in Indigenous maternal and infant health and well-being outcomes could be viewed as reflecting these problems. Indigenous women continue to experience unacceptably poorer pregnancy and birth outcomes overall (ARACY, 2018) and experience the highest rates of psychological distress compared to all other Australians (Australian Bureau of Statistics, 2019). However, they are four times less likely to receive perinatal mental health screening than non-Indigenous women. Those who are screened are three to four times more likely to be overlooked as being at high risk compared with non-Indigenous women (Gausia *et al.*, 2013), and when they are flagged, frequently no further action is taken (Langham *et al.*, 2017).

The negative trajectory in the health and well-being outcomes of Indigenous mothers and infants is likely to continue unless culturally safe and effective early identification strategies and interventions are explored, developed, and implemented. To this end, the aim of the study was to uncover the limitations of usual practice in Indigenous contexts. Instead, decolonizing methodologies prioritize Indigenous peoples’ knowledges and ways of addressing issues of concern to them.

### Decolonizing methodology

In her influential work, Māori academic Linda Tuhiwa Smith (2012) documented the critical importance of validating Indigenous knowledges through research that encompasses decolonization, healing, mobilization, and transformation. In his seminal text, Martin Nakata (2007) describes in detail the interface between Indigenous and scientific knowledge as an intersection where Indigenous researchers can generate new approaches using different lenses to non-Indigenous researchers. Ryder and colleagues describe the weaving together of these two diverse knowledge systems and world views. The interface represents a dynamic and impactful process, empowering researchers to reimagine their approach to research methodology, prioritizing the voices and knowledge of Indigenous peoples (2020).

Whilst this study was conceptualized by a non-Indigenous scholar and midwife (first author), based on her previous clinical work and research in the Kimberley and Tanzania, at every stage throughout, she ensured Indigenous peoples provided leadership with their viewpoints being continually privileged. This approach was integral to the successful study outcomes. Kovach (2009) and Tuck (2009) argue that decolonizing research paradigms whilst conferring a shift of power must purposefully consider the evolving relationship between researchers and participants. As non-Indigenous researchers, power must be actively shared. Being mindful of being the ‘outsider among insiders’ and shifting awareness from ‘self and others’ to viewing the ‘self as the other’ (Marriott *et al.*, 2020) are key to successfully ‘weaving’ of world views. This can and should be challenging as a non-Indigenous researcher. It requires shifts in thinking which drive the efforts to gather and deepen knowledge. Adopting critical reflection, letting go of culturally predetermined certainties, and being prepared to negotiate with clients and communities can be emotionally challenging and leave one feeling vulnerable, powerless, or out of place. Yet being willing and able to work outside one’s own comfort zone is crucial (Walker *et al.*, 2014).

Essential in this study was the commitment to adopting decolonizing approaches in all aspects of the research, from its inception to its conclusion. This ensured integrity and engendered confidence in the resultant evidence base for clinical translation. Four aspects of research design were selected to support a decolonizing approach to this study: (i) Aboriginal participatory action research (APAR), (ii) codesign principles, (iii) yarning, and (iv) mixed methodologies.

APAR is designed to place at the centre and increase the Indigenous voice and self-determination in Indigenous research. Its intention is to legitimatize Indigenous knowledges and methodologies as authentic, rightful, and critical components of transformative research in the Indigenous context to build self-determination in communities. Researchers can be vulnerable to constraints of funding rounds or organizational work constraints which may limit effective and representative community consultation. In this study, an authentically wide collaboration was critical to ensure shared ownership of the research design, the questions asked, the analysis of the data, and addressing issues arising. Over 18 months, this widespread community consultation resulted in Indigenous peoples from more than 17 Clans/Nations being actively committed to this research. Results were generated through iterative reflective cycles, where researchers and participants collect and analyse data together, then collaboratively determine the action to be taken whilst at all stages Indigenous viewpoints were privileged.

#### Codesign principles

Codesign is a process that aims to combine both the lived experience and professional expertise to identify and create a successful outcome (Sanders and Stappers, 2014). In Indigenous contexts, codesign plays a vital role. It reminds service providers and governments that they should do things with, and not to Indigenous communities (Dreise and Mazurski, 2018). The process of codesign was considered less vulnerable to the marginalization of Indigenous ways of knowing by non-Indigenous researchers, academics, and medical and mental health stakeholders more used to Western research methods. In combination, APAR and codesign involved the community in the full production of this research, including planning, design, and management ensuring a collaborative developmental process. The ‘balance of rights and powers’ for Indigenous communities and other stakeholders was established and maintained (Bradwell and Marr, 2008) to develop solutions that address the complexity of identified problems in maternal healthcare.

This was operationalized by inviting Indigenous peoples on Nyoongar Boodjar (country) to be principal partners, specifically to be co-researchers, leaders in the governance structure, and primary participants. This ensured multiple and varied opportunities for community members to deeply participate in and shape the research process. Results were generated through iterative reflective cycles. Participants were involved in data collection and analysis and in determining the action to take to improve the health of Indigenous families (Baum *et al.*, 2006). Codesign principles were central to the methodology developed.

#### Yarning methodology

In terms of data collection, yarning as described by Bessarab and Ng’andu (2010) provided a robust method for culturally safe and accurate data collection with Indigenous peoples. Whilst semi-structured interviews and narrative enquiry are not new (Schmidt, 2004), yarning requires the researcher to develop a relationship that is accountable to the Indigenous participant whilst following protocols for establishing a culturally safe and relevant dialogue. Yarning as a research method involves four conversational types: (i) a social yarn that weaves in and out of (ii) a research topic yarn that may move into (iii) a therapeutic yarn or (iv) a collaborative yarn. In combination, these yarning types enable issues to be deeply explored (Bessarab and Ng’andu, 2010) with the flow contextualizing the tapestry of data for interpretation.

#### Mixed methods

A mixed methods approach was adopted given the preliminary nature of the study area. When an area of study is ill-defined, it is important not to rely on a single data source but to capture data in an exploratory (rather than confirmatory) fashion. In this instance, a process of triangulation of quantitative and qualitative data, together with data from diverse sources, provided greater confidence in interpretations through the examination of points of convergence and divergence from complementary sources (Teddlie and Tashakkori, 2012). Quantitative data were collected through an anonymous online survey and were contrasted with data from individual and group-based yarning. Similarly, multiple participant types (including mothers and fathers as well as midwives and healthcare professionals) were critical in ensuring that diverse voices were heard.

The foundational commitment to a consistent decolonizing approach provided an emergent innovative solution. The result is a social and emotional well-being (SEWB) wrap-around screening programme. It breaks new ground by combining a strength-based and therapeutic approach to assessment using culturally and safe gender-specific digitized rubrics for mothers and fathers.

### Operationalizing the decolonizing methodologies

**Figure 1.**
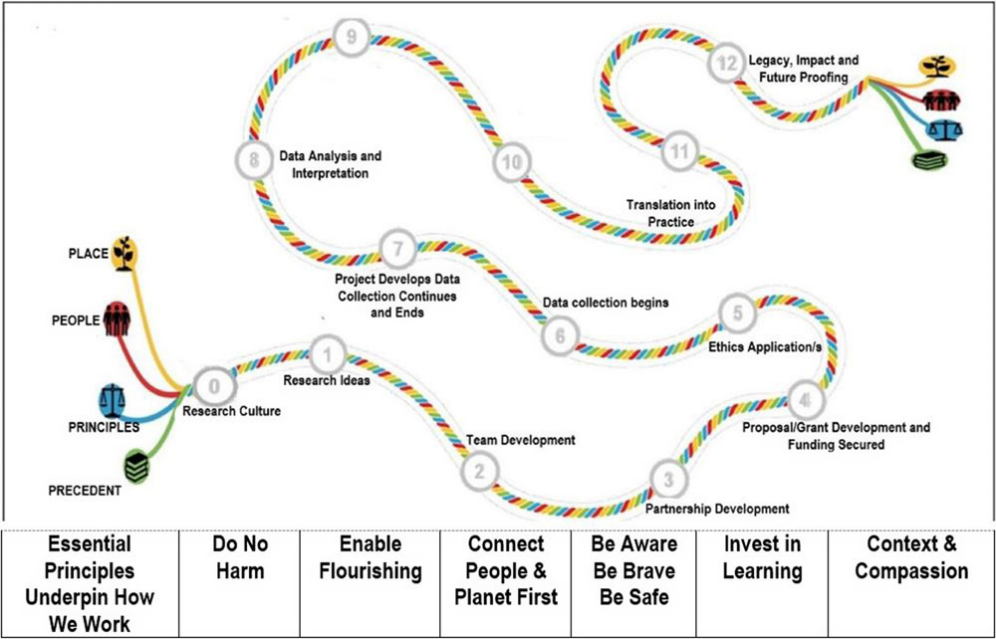
Stages and elements of an ethical research journey (Reproduced from Reid *et al.*, 2019; https://www.ethical-global-research.ed.ac.uk).

To be successful, application of decolonizing methodologies must be infused into the fabric of the project. Reid *et al.* (2021) suggest that re-iterative consideration of context and the involvement of key stakeholders at all stages of the research process are necessary for developing ethical outcomes when working in complex and cross-cultural contexts. They highlight in their ‘4P’s model of ethical research’ that any weak link in the research chain can constrain the validity of the entire project and compromise the clinical application of the findings. This is especially important in circumstances where inappropriate clinical application may result in harm to patients. Figure 1 outlines the 4P’s model of ethical research: place (context) and people need to be prioritized at each stage of the research journey along with a clear consideration of ethical principles and precedents (Reid *et al.*, 2021).

The comprehensive decolonizing approach adopted ensured that culturally safe processes enabled culturally safe, relevant, and translational outcomes. Operational examples of the decolonizing methodologies employed are described below.

#### Place

Understanding the context in which research is undertaken is critical in designing the optimal methodological approach. The cultural safety of the project was supported by ensuring that the research team had a suitable academic home. The Kalyakool Moort project was based at the Ngangk Yira Research Centre for Aboriginal Health and Social Equity at Murdoch University in Western Australia. Professor Rhonda Marriott, a senior Indigenous nurse and midwife and centre director was also the primary PhD supervisor of the first author (JK). Being anchored in an Indigenous research centre provided a rich environment and abundant academic and cultural support throughout the project. It also provided a safe welcoming space for Indigenous people to attend meetings.

**Figure 2.**
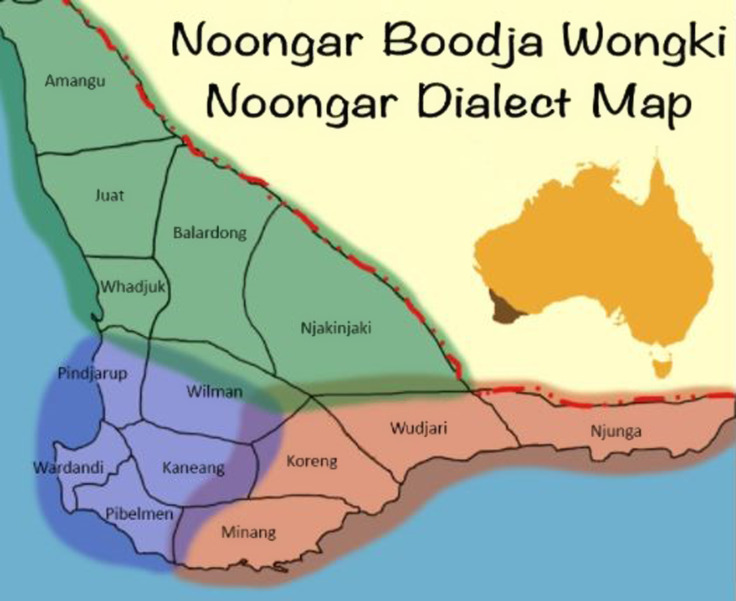
Map of Nyoongar Boodjar in Western Australia Reproduced from OLC Library Blogs (2015).

The geographical location for this study is Nyoongar Boodjar (country), which covers the Southwest of Western Australia and comprises metropolitan, regional, and rural communities (Figure 2). Fourteen clans make up the Nyoongar nation, and they predominate here as the Traditional Owners of the land. Recognizing that many Indigenous peoples from across Australia have made Nyoongar Boodjar their home, it was understood that some may feel dislocated from the country which may impact their mental health and well-being. Therefore, including participants with connections to diverse language groups, cultural practices or beliefs was important in establishing the Indigenous Advisory Groups. However, respecting the essential role Nyoongar Elders play on Nyoongar Boodjar in welcoming diversity whilst maintaining cultural safety and respect, was critical.

#### People

Predominant representation by Indigenous peoples throughout all aspects of the research was essential. Who was then involved in this collaborative effort was an important methodological step. JK spent the first 18 months of the study establishing relationships, building trust, and developing strong collaborations. Snowballing (Bonevski *et al.*, 2014) was used to engage Elders, senior Indigenous peoples, and community members. This included those working alongside Indigenous families and Indigenous and non-Indigenous service providers, managers, policymakers, researchers, and academics. This was essential in optimizing the codesign and translational capability of the research.

**Figure 3.**
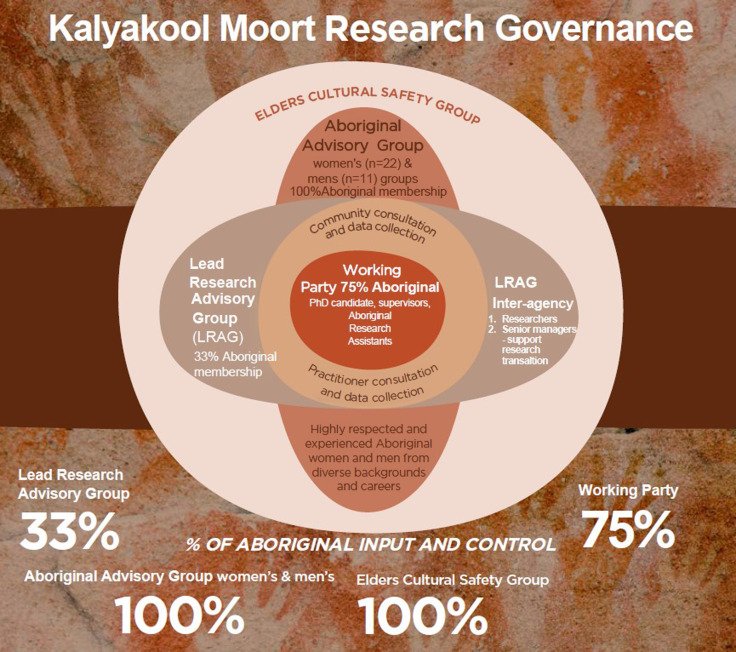
Kalyakool Moort Research Structure and Governance.

The study name ‘Kalyakool Moort’, meaning ‘always family’ in the Nyoongar language, the research hypothesis and questions, the governance framework (Figure 3), and the roles and responsibilities of the research team, were developed by Indigenous Elders and the Indigenous Advisory Group alongside JK.

#### Precedents

Understanding what has been done before is key to undertaking research ethically in complex environments (Reid *et al.*, 2021). This recognizes that research can be one-sided and burdensome for vulnerable and/or Indigenous communities. All research should ensure reciprocity, and it should not re-invent the wheel nor repeat mistakes. This entire study has involved learning from what has gone before by reviewing the literature and by interviewing experts with lived experience, specifically, Indigenous parents, and Indigenous and non-Indigenous perinatal health professionals.

#### Principles

**Figure 4.**
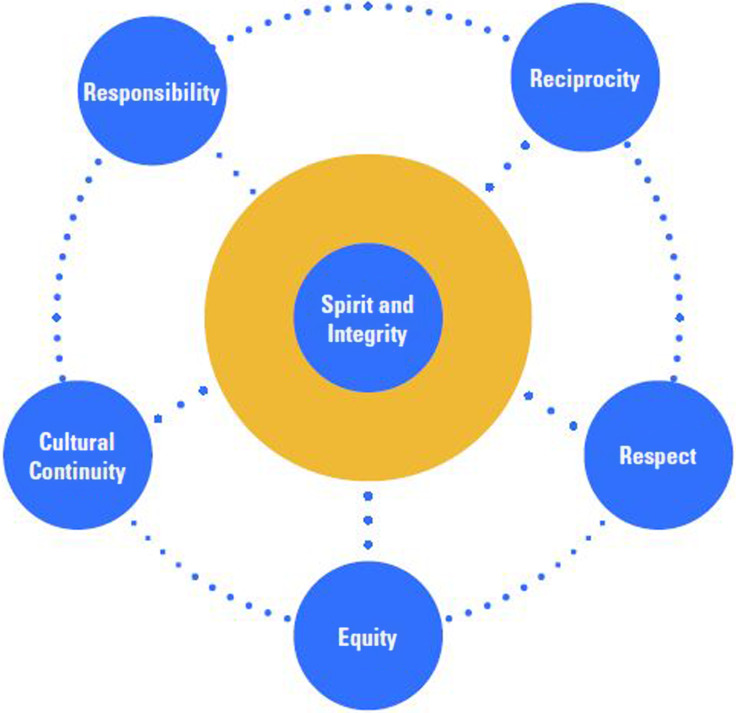
The six core values for ethical research conduct, important to all Aboriginal and Torres Strait Islander peoples (NHMRC, 2018).

The commitment to decolonizing research methodologies included compliance with all six principal ethical values (Figure 4) outlined in the National Values and Ethics: Guidelines for Ethical Conduct in Aboriginal and Torres Strait Islander Health Research (NHMRC, 2018). These values were operationalized and monitored by the Indigenous leadership and governance collective. Ethical approval was obtained from the Human Research Ethics Committee (HREC) at Murdoch University (approval 2013/202), the Western Australian Aboriginal Health Ethics Committee (approval 553), and the Women’s and Newborn Health Service HREC (approval HREC EC00350).

### Formulating the research inquiry

Indigenous Advisory Group workshops identified key issues for investigation. A yarning matrix was developed based on these key domains and provided a guide for the individual yarns and yarning circles (focus groups). The four Indigenous research assistants (two men, two women) received interrater reliability training in the use of yarning as a data collection strategy and dealing with disclosure. The data were collected within the Indigenous community. JK facilitated the focus groups and non-Indigenous professionals’ interviews. The anonymous 32-item online health professional questionnaire, informed by key issues identified in Indigenous Advisory Group workshops, was distributed through midwifery and child health networks across Western Australia and via the Kalyakool Moort website (www.alwaysfamily.weebly.com).

### Recruitment

The study employed purposive and snowball sampling with a distinctive cultural approach drawing on kinship, professional, and community connections to reach Indigenous participants. Community participants initially were identified through connections via the Indigenous Advisory Group and the Elders Cultural Safety Group members. This continued through local community advertising, word-of-mouth, and snowballing through multiple entry points across the region. The snowball sampling resulted in a diverse spread of participants’ opinions and experiences. Professionals were recruited via invitation through organizational networks.

### Consent

A fundamental ethical principle concerns the recognition of participants’ rights, including the right to be informed about the aims of the project, to freely decide whether to participate without coercion, and the right to withdraw without penalty at any time (NHMRC, 2018). Indigenous parents were invited to participate and given information about the study, with a separate occasion arranged in a relaxed environment for the yarns to take place. All participants consented to the digital recording of their yarns. The same applied to professionals; however, four professionals requested that recordings be stopped at sensitive points of discussion.

### Data transcription

Drawing on a naturalized transcription method, digital recordings were usually transcribed by the researcher undertaking the individual yarns or yarning circles. This approach maintained a cultural lens on the transcribed data, capturing nuances associated with low talking, inflections, silences, and body language (noted in field notes at the point of the interview), all important aspects in Indigenous peoples’ communication. For example, the word ‘good’ was used frequently to describe perinatal care. However, the meanings were vastly different. Audio inflections of the term ‘good’ were easily identified as being ‘extremely poor’ or ‘alienating’ experiences, to ones of ‘excellent’ care. All such nuances were noted by the researcher/transcriber. Overall, the triangulation of results from detailed thematic analysis of individual yarns with Indigenous parents and professionals was reinforced by results from community and professional workshops, thus increasing the trustworthiness of data outcomes and enhancing the richness and strength of findings. The results from the health professional online survey further strengthened confidence in emergent themes.

### Data analysis

Data analysis was carried out drawing on thematic analysis and Charmaz’s (2007) approach to grounded theory. Here an inductive and comparative approach to thinking and analysis, alongside data collection and analysis occurring simultaneously, ensured researchers remained constantly involved in the emerging data analysis. This required being aware that changing context or perspectives of reality impact analysis and that personal knowledge, in this case, Indigenous peoples’ knowledge and experience aided the knowing (Singh and Estefan, 2018). The data analysis moved from the specific to general and then to specific conclusions. Audios were first listened to, then transcribed, read, re-read, and then coded by each individual investigator. This ensured that initial coding was informed by first-hand cultural knowledge. The research team (80% Indigenous) also individually listened to, read, and re-read transcripts, coding concepts, and themes. Concepts were then discussed as a group and themes crystallized using inductive-deductive, comparative, interactive, and iterative strategies until consensus was reached. This upheld the fidelity of analysis and reduced interpretive bias. Emergent key themes were further explored and relationships, consistencies, and incongruities with the existing literature were examined. NVIVO © 2020 was used to aid analysis by providing an additional and more manageable way of coding data and considering relationships.

### Data interpretation

Research findings were routinely reported back to the Indigenous Advisory Group and then the Elders Cultural Safety Group for ongoing reflection, to inform and be informed by the unfolding themes. Themes once refined to those most significantly associated with the research questions were categorized as either a barrier or enabler to (i) strong parenting practices or (ii) culturally responsive and effective screening practices. Over many workshop sessions, Indigenous Advisory Group members evaluated and brainstormed solutions to each. The Research Group, followed by the Elders Cultural Safety Group, assessed and made further modifications or solutions.

**Figure 5.**
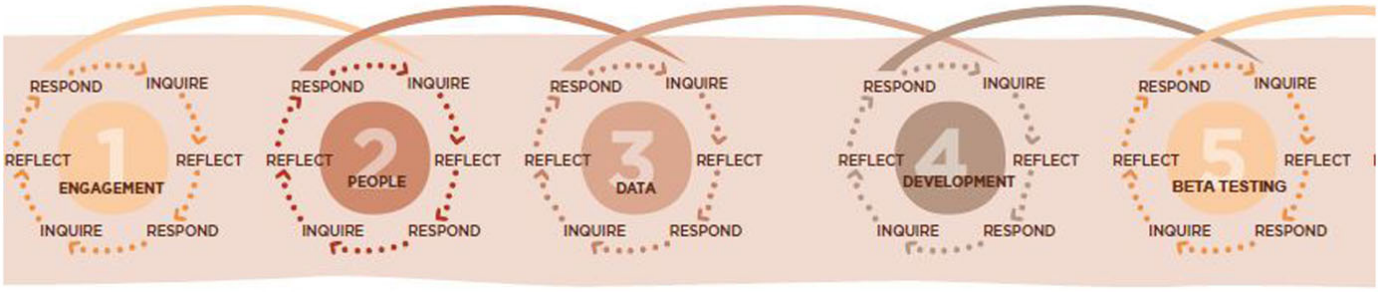
Iterative process of generating solution through codesign.

This iterative process continued, as the process progressively codesigned a set of wrap-around recommended solutions (Figure 5). Funding was incrementally sought to implement these recommendations, resulting in an innovative digital platform called the *Baby Coming You Ready* programme.

### Clinical application

This highly iterative process of taking evidence gathered and translating it into clinical practice recommendations and tools has been with and for the benefit of Indigenous communities and professionals working alongside them. The emergent body of work is an innovative strengths-based holistic perinatal assessment with therapeutic potential. This wrap-around and strengths-based programme called *Baby Coming You Ready* (BCYR) centres around a digital assessment on iPads that uses Indigenous voiceovers and touchscreen images to guide a reflective clinical yarn (narrative inquiry) in perinatal clinical settings.

## Discussion

There is ample evidence that perinatal mental health screening with Indigenous mothers is not achieving the desired outcomes. The Closing the Gap Prime Ministers Report (2016) highlights the perinatal period as providing an important window of opportunity for primary care providers to implement effective perinatal health and well-being strategies (Department of the Prime Minister and Cabinet, Australia, 2016). The National Aboriginal and Torres Strait Islander Health Plan 2013–2023 stresses that healthcare be underpinned by culture so that

*a healthy, safe and empowered life with a healthy strong connection to culture and country…receiving care based on the best possible evidence…free of racism and inequality…that individuals and communities actively engage in decision making and control…and that mother and babies get the best possible care and support for a good start to life.* (Australian Government, 2013)

Consciously adopting APAR as a decolonizing methodology (Dudgeon *et al.*, 2020) throughout the 18-month consultation and development period of Kalyakool Moort saw trust in relationships strengthen and power consciously shared, as Indigenous collaborators frequently voiced ‘nothing about us, without us’. Indigenous peoples became researchers. Recognizing the benefits of power-sharing, all are jointly committed to co-creating and facilitating the Kalyakool Moort research. The codesigned solution drew heavily on Indigenous peoples’ experiences and wisdom alongside the collective experiences of Indigenous and non-Indigenous stakeholders (researchers, midwives, child health nurses, psychologists, general practitioners, mental health consultants, and social workers).

APAR incorporates similar principles to codesign models of research incorporating high levels of community consultation and engagement (Dudgeon *et al.*, 2020) as was the case with Kalyakool Moort. Researchers and other key stakeholders were required to concede perceptions of power, influence, and pre-conceived agendas. This resulted in a progressive translational solution that the Aboriginal community could own.

Authentic community engagement is crucial to successful sustainable interventions that support effective perinatal primary health care. Unquestionably codesign is a preferred approach to decolonizing research and to developing solutions with and therefore for Indigenous peoples, communities, and key stakeholders. This study confirmed that this approach requires strong collaboration between researchers and all end users from the outset. It includes jointly developing questions, deciding on research design, and influencing implementation and broader dissemination strategies (Goodyear-Smith *et al.*, 2015).

Whilst the codesign model was critical to success, it also presented challenges. Workplace, academic and funding constraints and time limits, and restrictive requirements set by universities and HRECs were at times at loggerheads with the evolving and adaptive processes central to this codesigned research. Institutions require precise pre-definitions of methodologies, objectives, strategies, interventions, costings, and outcome measurements to protect participants from harm, to support rigour and transparency, and to ensure financial accountability. However, detailing all prerequisites prior to the phases of codesign research is not always feasible nor reflective of strong practice. To remain true and accountable to the codesign process whilst preventing shortcuts, commitment, patience, flexibility, and resourcefulness were required. The complex cultural interface of this research, at times, presented challenges. Being both attuned and responsive to these challenges was critical. Research in perinatal mental health with Indigenous peoples necessitates emersion in the Indigenous worldview SEWB (Dudgeon *et al.*, 2020; Gee *et al.,* 2014). This is both multidimensional and complex, deeply rooted in intergenerational connection to land or ‘country’, spirit, family, kinship connections, ancestors, and community. It is also enmeshed in complex trauma but counterbalanced with an innate resilience both at individual and community levels (Usher *et al.*, 2021).

Despite nominal recognition of the importance of SEWB factors in the Western healthcare system, there remains an enduring focus on mental illness and psychopathology with most professionals holding fast to the medicalized understanding of mental illness. This may in part be due to an understandable fear of ‘missing’ the worst-case scenario, suicidal intent. However, failing to appreciate SEWB from Indigenous peoples’ worldview perpetuates cultural ignorance and fear of the consequences of being misjudged and misdiagnosed. With specific regard to perinatal mental health screening, without understanding the importance of SEWB within the complex context of Indigenous peoples’ lives, clinicians run the risk of missing early red flags. This further alienates mothers and families most in need of support.

Other cultural complexities arose from the interdisciplinary nature of this research. Perinatal mental health care rarely involves one discipline, as each brings a unique perspective to the shared goal. Interdisciplinary collaboration and input were critical. However, most professionals, being trained and socialized into their own professions, brought in biased understandings of mental health, of SEWB, and of their own roles and the roles of others. These professionally bound biases were often in opposition to the importance of Indigenous perceptions of SEWB. Overall, establishing a research framework in which Indigenous people guided the process and retained privilege was definitive in achieving a successful outcome. BCYR has emerged as an evidence-based primary healthcare assessment rubric and intervention with the potential to transform how Indigenous women are able to participate in culturally safe and meaningful perinatal mental health screening and supportive follow-up.

### Conclusion

The transference of existing screening tools and processes, originally adapted for the mainstream population, onto the Indigenous peoples is not working (Kotz, 2021). It is doubtful any method will be culturally responsive and effective unless the approach to screening and to follow-up strategies are community driven. The decolonizing approach taken in this project has been committed to codesign and to community leadership and ownership of the process from the beginning. The project’s success was strengthened by maintaining both a desire-based and strength-based approach to codesigning the outcome. Factors influencing its success include the maintenance of the research team and advisors who were professionally diverse (midwifery, psychology, psychiatry) and culturally diverse (Indigenous peoples from various clans and nations) and agreement on outcomes of thematic analysis across all stakeholder groups. Whilst time-consuming in the short term, the project identified an innovative solution with long-term sustained positive maternal and child health outcomes. The BCYR programme is a highly successful and translational research outcome emerging from decolonizing research and pilot evaluation in real-world clinical settings.

## Footnotes

1 In this publication, the term *Indigenous peoples* is used when referring to Aboriginal and Torres Strait Islander people. The use of the plural *peoples* is used to acknowledge the cultural diversity within all Aboriginal and Torres Strait Islander communities.
